# Foraging behavior links sea ice to breeding success in Antarctic penguins

**DOI:** 10.1126/sciadv.aba4828

**Published:** 2020-06-24

**Authors:** Yuuki Y. Watanabe, Kentaro Ito, Nobuo Kokubun, Akinori Takahashi

**Affiliations:** 1 National Institute of Polar Research, Tachikawa, Tokyo 190-8518, Japan.; 2 Department of Polar Science, The Graduate University for Advanced Studies, SOKENDAI, Tachikawa, Tokyo 190-8518, Japan.

## Abstract

Population trends and breeding success variability of Adélie penguins, a bioindicator of Antarctic environments, have been attributed to changing sea-ice extents; however, causative mechanisms remain unclear. By electronically tagging 175 penguins in four seasons with contrasting sea-ice conditions, we show that ice-free environments enhance, not deteriorate, foraging efficiencies and breeding success. In an ice-free season, penguins traveled by swimming rather than walking, leading to larger foraging areas, shorter trip durations, and lower energy expenditure than three ice-covered seasons. Freed from the need to find cracks for breathing, dive durations decreased, and more krill were captured per unit dive time, which may also be associated with phytoplankton blooms and increased krill density in the sunlit ice-free water. Consequently, adult body mass, chick growth rates, and breeding success increased. Our findings explain the regional population trends and demonstrate a key link among sea ice, foraging behavior, and reproductive success in this iconic species.

## Teaser

In ice-free summer, Antarctic penguins enjoy easy access to food and increase body weight and breeding success.

## INTRODUCTION

In polar regions, different seascapes can appear from year to year due to interannual variabilities in sea-ice extents. During the last few decades, sea-ice extents have markedly decreased in the Arctic, whereas they have, counterintuitively, increased in Antarctica (*1*). Such changes in sea ice are expected to alter the habitats of marine organisms physically (e.g., by changing areas where penguins, seals, and polar bears can walk, swim, or rest) and biologically (e.g., by changing sunlight penetration and primary production). Toward understanding the ecosystem-level impacts and predicting future changes, we need to understand the fitness consequence of changing sea ice in ecologically important model organisms such as seabirds.

Adélie penguins, *Pygoscelis adeliae*, are abundant, ice-dependent (*2*) seabirds with a circum-Antarctic continental distribution. As an important bioindicator of the Southern Ocean ecosystem, population trends and breeding success of Adélie penguins and their association with sea-ice conditions have been intensively studied (*3*–*11*). The results vary among studies. In the continental Antarctic region, breeding success increases (*5*) and populations start growing (*6*–*8*) in the years of sparse sea ice. Similarly, massive breeding failures occurred in the years with unusually extensive sea ice (*9*). By contrast, Adélie penguin populations in the maritime Antarctic region (the Antarctic Peninsula and adjacent islands, including South Orkney and South Sandwich Islands) are negatively affected by decreasing sea-ice extent (*10*, *11*). More recently, both breeding success (*12*) and survival rates during the winter (*13*) were shown to peak at modest sea-ice extents [i.e., optimal sea-ice model (*14*)]. These mixed results suggest that sea ice can have either positive or negative effects on Adélie penguin survival and breeding success through a combination of mechanisms. For example, extensive sea ice may limit the accessibility to waters and, thus, foraging activities (*5*–*7*), whereas extensive sea ice in the winter is thought to enhance the recruitment of Antarctic krill (*15*), the main prey of Adélie penguins (*16*). Crucially, most of the previous studies are correlational rather than experimental, and mechanistic links between sea-ice extent and breeding success remain unclear. A missing piece of information is foraging behavior, which could directly link changing environments to breeding success in seabirds (*17*). Although several studies recorded simple metrics of Adélie penguin foraging behavior (e.g., trip duration, dive depth, and dive duration) over multiple breeding seasons with different sea-ice conditions (*12*, *16*, *18*), no clear links have been found.

**Fig. 1:**
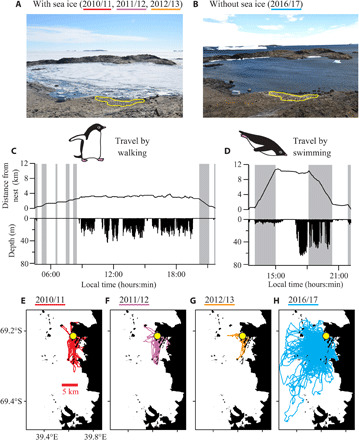
Sea-ice conditions and penguin behavior. Scenes around the penguin colony studied (yellow line) photographed in (**A**) January 2012, when sea ice was extensive, and (**B**) January 2017, when sea ice was nearly absent (Photo credit: Y.Y.W., National Institute of Polar Research). Typical foraging trips of penguins recorded in (**C**) ice-covered January 2012 and (**D**) ice-free January 2017, showing distance from nest and dive depth. Penguins traveled by walking and swimming in 2012 and 2017, respectively, during the periods denoted by gray bars. The scales of both the *x* and *y* axes are matched in (C) and (D) to facilitate comparison. (**E** to **H**) All foraging trips recorded by GPS loggers for each season. Yellow circles represent the colony.

Here, we show such mechanistic links by recording Adélie penguin foraging behavior in the greatest detail yet by using animal-borne Global Positioning System (GPS) loggers, accelerometers, and video cameras in four breeding seasons (*N* = 175 birds). These modern devices allowed us to track foraging trips, categorize behavior (e.g., walking, swimming, and resting) (*19*), and estimate the number of prey captured during dives (*20*). Our study site, located in Lützow-Holm Bay, continental East Antarctica, is characterized by permanent fast sea ice that covers the entire foraging areas of breeding penguins (Fig. 1A). However, one of the four seasons (2016/17) was unusual in that a large portion of fast sea ice broke up in the bay and was carried away by currents in the last autumn, an event that has occurred six times since the 1950s (*21*). Although new sea ice formed after that, an extensive polynya was present around the penguin colony throughout the breeding season (Fig. 1B; see also fig. S1 for satellite images). This unexpected “natural experiment” revealed how changing sea ice alters penguin foraging behavior and, consequently, their body condition and breeding success.

## RESULTS

**Fig. 2:**
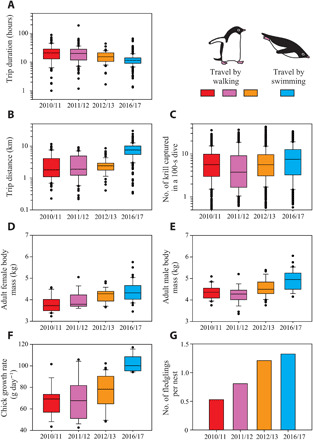
Interannual variability in penguin performance. (**A**) Trip duration, (**B**) trip distance (i.e., the maximum distance from nests reached during the trips), and (**C**) the number of krill captured in a dive, standardized for a 100-s dive duration, plotted on a log scale. Body mass of adult (**D**) females and (**E**) males, (**F**) chick growth rates, and (**G**) the number of fledglings per nest, plotted on a linear scale. In box plots, the 25th percentile, the median, and the 75th percentile are shown by boxes, the 10th and 90th percentiles are shown by error bars, and the data points outside the 10th and 90th percentiles are shown by small circles.

GPS tracking data (*N* = 87 birds), part of which also had accelerometer records (*N* = 37 birds), showed different penguin behaviors among seasons. In the ice-covered seasons (2010/11, 2011/12, and 2012/13), penguins walked (and sometimes tobogganed) on the sea ice or along the coast after leaving their nests, with occasional resting behavior that lasted for minutes to hours, and then found cracks in the ice where they dived repeatedly (Fig. 1C and movie S1). In the ice-free season (2016/17), by contrast, penguins entered the water directly in front of their nests, swam offshore with repeated shallow (<10 m) dives or mixed shallow and deep dives, and then conducted a series of deep foraging dives (Fig. 1D); however, many individuals also rested on land (e.g., islands) or small pieces of floating sea ice during portions of their trips. The foraging areas expanded (Fig. 1, E to H), and diving locations were not limited by the location of cracks in the ice-free season. On the basis of linear mixed-effect models, season affected both the durations [likelihood ratio test, χ^2^(3) = 23.3, *P* < 0.0001] and distance of foraging trips [χ^2^(3) = 62.4, *P* < 0.0001], with the ice-free season having, on average, 3.2- to 7.9-hour (21 to 40%) shorter duration and 4.5 to 4.8 km (188 to 229%) longer distance compared with the three ice-covered seasons (Table 1 and Fig. 2, A and B). The shorter duration combined with the longer distance is possible because penguins traveled faster when swimming (mean ± SD, 5.6 ± 0.6 km hour^−1^, *N* = 18 events) than walking (1.5 ± 0.6 km hour^−1^, *N* = 84 events). The effect of locomotory modes (walking or swimming) was also evident in the relationship between trip distance and trip duration (fig. S2), which showed that the ice-free season had by far the shortest duration for a given distance. To examine the energetic consequence of this change, a subset of GPS tracking data that also had accelerometer records (*N* = 37 birds) was analyzed. The proportions of time spent in walking, diving, resting at the sea surface, and resting on land or ice during the trips were highly variable among individuals, except that penguins spent little time walking in the ice-free season (fig. S3). Because of this variability, the total energy expenditure during trips, estimated from the time spent in each activity and activity-specific metabolic rates previously reported (Materials and Methods), was largely determined by trip duration alone regardless of the presence or absence of sea ice (fig. S4). Therefore, foraging trips of shorter duration observed in the ice-free season were energetically efficient.

**Table 1:** Foraging and breeding performance of Adélie penguins in four seasons. Values shown are estimates (with 95% confidence interval) based on linear mixed-effect models for foraging behavior and means ± SD or single observations for others.

	**2010/11**	**2011/12**	**2012/13**	**2016/17**
Sea-ice cover	Yes	Yes	Yes	No
Foraging behavior				
Trip duration (hours)	19.3 (15.9–23.7)	19.7 (12.4–31.4)	15.0 (9.0–25.1)	11.8 (7.6–18.4)
Trip distance (km)	2.1 (1.6–2.7)	2.4 (1.4–4.3)	2.4 (1.3–4.5)	6.9 (4.1–11.9)
	(*N* = 91 trips from 15 birds)	(*N* = 92 trips from 28 birds)	(*N* = 64 trips from 17 birds)	(*N* = 260 trips from 27 birds)
Dive duration intercept (s)*	69 (58–80)	74 (48–100)	81 (56–106)	54 (30–79)
	(*N* = 531 dives from 8 birds)	(*N* = 485 dives from 9 birds)	(*N* = 1119 dives from 15 birds)	(*N* = 2275 dives from 19 birds)
No. of krill captured in a 100-s dive	4.1 (2.8–6.1)	3.6 (1.4–9.2)	5.2 (2.2–12.6)	5.4 (2.2–13.2)
	(*N* = 443 dives from 8 birds)	(*N* = 374 dives from 9 birds)	(*N* = 996 dives from 15 birds)	(*N* = 1211 dives from 12 birds)
Body condition				
Adult female body mass (kg)	3.8 ± 0.4 (*N* = 21 birds)	4.0 ± 0.4 (*N* = 18 birds)	4.2 ± 0.3 (*N* = 29 birds)	4.4 ± 0.5 (*N* = 42 birds)
Adult male body mass (kg)	4.4 ± 0.3 (*N* = 21 birds)	4.2 ± 0.4 (*N* = 28 birds)	4.6 ± 0.4 (*N* = 42 birds)	4.9 ± 0.5 (*N* = 31 birds)
Breeding status				
Chick growth rate (g day^−1^)	67 ± 14 (*N* = 14 birds)	69 ± 20 (*N* = 15 birds)	76 ± 16 (*N* = 33 birds)	102 ± 8 (*N* = 18 birds)
No. of nests (in late December)	70	109	181	108
No. of fledglings (in early February)	37	88	219	143
Stomach content				
Prey composition by mass†	Krill 38 ± 46%	Krill 37 ± 46%	Krill 91 ± 19%	Krill 94 ± 14%
	Fish 62 ± 46%	Fish 63 ± 46%	Fish 9 ± 19%	Fish, 6 ± 14%
Krill composition by mass	*E. superba* 32 ± 24%	*E. superba* 73 ± 42%	*E. superba* 81 ± 34%	*E. superba* 100%
	*E. crystallorophias* 68 ± 24%	*E. crystallorophias* 27 ± 42%	*E. crystallorophias* 19 ± 34%	
	(*N* = 10 birds)	(*N* = 10 birds)	(*N* = 11 birds)	(*N* = 8 birds)

*Intercept of dive duration as a function of dive depth (i.e., dive duration of a hypothetical 0-m dive).

†In addition to krill and fish, a few amphipods (<1% by mass in most birds) were often found.

**Fig. 3:**
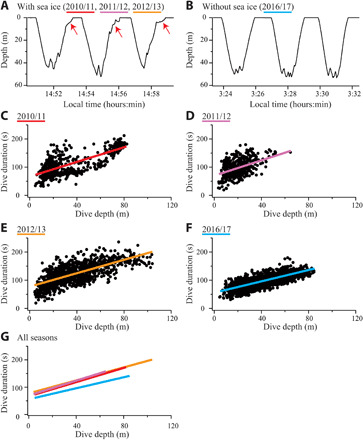
Penguin diving behavior. Typical krill-feeding dives recorded in (**A**) January 2013, when sea ice was extensive, and (**B**) December 2016, when sea ice was nearly absent. In ice-covered seasons, penguins slowed ascending speeds down at the end of each dive to search for cracks in the ice for breathing [red arrows in (A)]. (**C** to **G**) Relationships between dive depth and dive duration of krill-feeding dives for each season, with the ordinary least-squares regression lines (2010/11, *Y* = 1.30 × *X* + 66.6, *R*^2^ = 0.57, *N* = 531; 2011/12, *Y* = 1.36 × *X* + 69.7, *R*^2^ = 0.20, *N* = 485; 2012/13, *Y* = 1.19 × *X* + 77.3, *R*^2^ = 0.49, *N* = 1119; 2016/17, *Y* = 1.01 × *X* + 55.7, *R*^2^ = 0.60, *N* = 2275).

The analyses of foraging dives based on accelerometer records (*N* = 51 birds) showed that, in the ice-covered seasons, penguins often slowed ascending speeds down at the end of dives, indicating that they searched for and moved to cracks for breathing (Fig. 3A). In the ice-free season, by contrast, penguins ascended straight to the surface from the depths where they foraged (Fig. 3B). As a result, season affected dive durations [χ^2^(3) = 20.7, *P* < 0.0005], with the ice-free season having, on average, 15- to 27-s (22 to 33%) shorter duration (as assessed by the intercept of dive duration as a function of dive depth) compared with the three ice-covered seasons (Table 1 and Fig. 3, C to G). Dive durations were also less variable for a given depth in the ice-free season (*R*^2^ = 0.60) compared with the ice-covered seasons (*R*^2^ = 0.20 to 0.57). The difference in diving behavior was even more evident when each dive was separated into descent, bottom, and ascent phases (Materials and Methods). Descent phases had similar duration with similar variabilities for a given depth for all seasons, whereas ascent phases had shorter and less variable duration in the ice-free season compared with the ice-covered seasons (fig. S5). Thus, shorter and less variable dive durations observed in the ice-free season are primarily due to the difference in ascent phases, during which penguins were freed from the need to find cracks in the ice.

The number of krill captured in a dive (standardized for a 100-s dive) (*N* = 44 birds), estimated from penguin head accelerations relative to body accelerations (*20*), was highly variable for each season. It was, on average, 0.2 to 1.8 individuals (4 to 50%) higher in the ice-free season compared with the three ice-covered seasons (Table 1 and Fig. 2C), although the effect of season was statistically nonsignificant [χ^2^(3) = 3.6, *P* = 0.31]. Water was blue in the ice-covered seasons but green in the ice-free season, as seen in the penguin-borne video footage (fig. S6). This observation agreed with unusually high chlorophyll a concentrations (23.8 to 38.6 mg m^−3^, *N* = 4) measured in the ice-free season for the surface water sampled by a ship 17 to 21 km off the penguin colony.

Season affected body mass of adult females [analysis of variance (ANOVA), *F*_3,106_ = 10.6, *P* < 0.0001] and males (*F*_3,118_ = 13.7, *P* < 0.0001) and the growth rates of chicks (*F*_3,76_ = 20.09, *P* < 0.0001), with the ice-free season having, on average, 0.2 to 0.6 kg (5 to 16%) larger female body mass, 0.3 to 0.7 kg (7 to 17%) larger male body mass, and 26 to 35 g day^−1^ (34 to 52%) higher chick growth rate compared with the three ice-covered seasons (Table 1 and Fig. 2, D to F). Breeding success, defined as the number of fledglings per nest, was also highest in the ice-free season (Fig. 2G), although the absolute number of nests and fledglings were highest in an ice-covered season (2012/13) (Table 1).

Stomach content analysis (*N* = 39 birds) showed that Antarctic krill *Euphausia superba* was the single dominant prey (94% by mass) in the ice-free season. By contrast, two krill species (*E. superba* and *Euphausia crystallorophias*) and fish (mostly *Pagothenia borchgrevinki*) were found in different proportions in the three ice-covered seasons (Table 1).

## DISCUSSION

We showed that Adélie penguins enjoyed favorable foraging conditions in the ice-free season at two spatiotemporal scales (i.e., foraging trip scale and individual dive scale). At the foraging trip scale, the most important change was that penguins traveled by swimming rather than walking. Because swimming is four times faster than walking, penguins searched larger areas for prey in shorter durations in the ice-free season (Figs. 1, E to H, and 2, A and B). This change is not only time efficient but also energy efficient, because total energy expenditure during the trips was largely determined by trip duration alone [fig. S4, see also (*22*)]. Given the estimates of trip duration based on linear mixed-effect models (Table 1) and the relationship between trip duration and energy expenditure (fig. S4), penguins may have expended 381 kJ kg^−1^ per trip on average in the ice-free season, which is 15 to 33% less than the 450 to 572 kJ kg^−1^ estimated for the ice-covered seasons. Energetically efficient foraging trips in the ice-free season can also partly be explained by the cost of transport (i.e., the energy required to move a unit body mass over a unit distance), which is four times lower for swimming (7 J kg^−1^ m^−1^, assuming 14.0 W kg^−1^ at 2.0 m s^−1^; Materials and Methods) than walking (26 J kg^−1^ m^−1^, assuming 10.7 W kg^−1^ at 1.5 km hour^−1^) in penguins. Moreover, penguins did not need to find cracks in the ice in the ice-free season but instead dived anywhere they wanted. This advantage likely helped penguins to further save time and to mitigate intraspecific competition for prey. In colonial breeding seabirds, intraspecific competition for prey is so severe that it can cause local prey depletion (*23*) and influence population trajectories (*24*). Antarctic penguins diving through spatially limited sea-ice cracks, such as Adélie penguins in our study site in normal sea-ice conditions, may represent an extreme case of intraspecific competition.

At the individual dive scale, the ice-free condition freed penguins from the need to find cracks for breathing at the end of each dive, leading to shorter and less variable ascent duration (fig. S5) and total dive duration (Fig. 3). For example, a foraging dive to 50-m depth with the ascent phase starting at 40 m required, on average, a breath-hold for 106 s including 34-s ascent phase in the ice-free season. These values are 20 to 23% and 39 to 47% shorter than the dive duration (132 to 138 s) and ascent duration (56 to 64 s), respectively, required in the ice-covered seasons. As dive duration increases above a certain level, disproportionally longer postdive surface duration is required to replenish oxygen stores in the body, leading to decreased proportion of foraging duration in a dive cycle (dive duration plus the subsequent surface duration) (*25*). Therefore, shorter and less variable dive durations recorded in the ice-free season impose less physiological stress on Adélie penguins and help them to forage more efficiently.

Furthermore, phytoplankton bloomed in the ice-free season, as evident from visually green waters (fig. S6) and high chlorophyll a concentrations (>23 mg m^−3^) recorded off the penguin colony. In the Antarctic coastal waters with seasonal ice cover, phytoplankton growth is limited by sunlight and the loss of sea ice is followed by intensive phytoplankton blooms (*26*). Coupled with our observation that Antarctic krill *E. superba* was the single dominant prey of penguins in the ice-free season (Table 1), we postulate that the phytoplankton bloom caused by the loss of sea ice may have attracted *E. superba* and increased its local density. *E. superba* prefers both oceanic and coastal waters with high chlorophyll a concentrations in the Southern Ocean (*27*), whereas *E. crystallorophias* (another krill species found in the ice-covered seasons) prefers high-latitude, coastal habitats with heavy ice cover (*28*). A combination of shorter dive durations and potentially increased local krill density in the ice-free season is highlighted by the highest number of krill captured per unit dive time in that season (Fig. 2C). *E. superba* (reaching 54 mm in length) is much larger than *E. crystallorophias* (reaching 36 mm in length) (fig. S7) (*29*). Although krill species cannot be identified from the tag data, our observations suggest that the energy-based foraging efficiency (i.e., energy gained per unit dive time) would be pronounced in the ice-free season.

The favorable foraging conditions in the ice-free season, both at the foraging trip scale and individual dive scale, were associated with increased adult body mass, chick growth rates, and breeding success (Fig. 2, D to G). This result is not a coincidence but a direct causal link, because foraging success and chick-feeding frequency (determined by trip duration) dominantly affect chick growth and survival as well as adult body conditions in seabirds (*30*, *31*). In support of this view, when the three ice-covered seasons were compared, the 2012/13 season, which had the shortest trip durations and highest krill capture rates, was associated with the highest adult body mass, chick growth rates, and breeding success (Table 1 and Fig. 2). Variable breeding success within the three ice-covered seasons also stresses that the presence or absence of sea ice is not the only factor determining penguin foraging conditions or breeding success. Moreover, the highest number of nests at the beginning of the breeding season and, consequently, the highest absolute number of fledglings at the end of the breeding season were recorded in the ice-covered 2012/13 season rather than in the ice-free 2016/17 season. This result supports the idea that population sizes in summer breeding season are also affected by the conditions in winter (*6*, *13*). Despite such complexities, our results demonstrate a clear mechanistic link among sea-ice extent, foraging behavior, and breeding success in Adélie penguins, an iconic polar predator.

Our finding explains the previous analyses on the Adélie penguin populations in the continental Antarctic region (including our study site), where breeding success and population growth rates are negatively affected by increasing sea-ice extents (*5*–*8*). More broadly, our natural experiment provides mechanical explanations to the optimal sea-ice model, in which decreasing sea-ice extents increase penguin fitness in areas where sea ice is normally extensive (*12*–*14*). Climate models predict a rapid reduction in the Antarctic sea-ice extents over the 21st century (*32*), despite the opposite long-term trend currently being observed. A recent study reported rapid reductions in Antarctic sea ice in the last few years, with the year 2017 having the lowest yearly average in the 1979–2018 record (*33*). Therefore, Adélie penguin populations in the continental Antarctic region are likely to grow in the coming decades. A poorly understood, important factor is how local density and species composition of krill might change with long-term trends of sea-ice extents. However, this is not the whole story, because Adélie penguin populations in the warmer, maritime Antarctic region, which represent approximately 30% of the total population of the species (*34*), apparently show the opposite, negative response to decreasing sea-ice extents (*10*, *11*). We postulate that the mechanism found in this study (i.e., penguins travel and dive more efficiently in open waters than in ice-covered waters) still plays a role, but it is overridden by other factors, most likely prey availability and behavior-specific energy expenditure, in that region. For example, if too little sea ice precludes penguins from resting on ice while they are offshore, extra thermoregulation cost incurred in water compared to in air (8.4 W kg^−1^ versus 4.8 W kg^−1^; Materials and Methods) could deteriorate their energy balance. The penguins tagged in this study rested for extended periods exclusively on land or ice, rather than at the sea surface, during foraging trips. They never stayed at the sea surface for >5 min in the ice-covered seasons (in the 613-hour record composed of 30 trips) and did so only rarely (10 times in the 197-hour record composed of 14 trips) even in the ice-free season. To understand the case where sea ice is normally sparse, detailed biologging studies on foraging behavior and activity time budget, as demonstrated in this study, are warranted for penguin populations in the maritime Antarctic region.

In conclusion, we show that ice-free environments allow Adélie penguins to travel by swimming rather than walking and to dive freely without the need to find cracks in the ice. These changes lead to larger foraging areas, shorter trip durations, and lower energy expenditure during the trips and potentially mitigate intraspecific competition for prey. More krill are captured per unit dive time, which may be associated with phytoplankton blooms and increased krill density caused by direct sunlight exposure of the water. As a result, counterintuitively for this ice-dependant species, body conditions and breeding success improved in the ice-free environment. The mechanistic link among sea-ice extent, foraging behavior, and breeding success found in this study helps us to explain the regional population trends of the species and predict future changes, although other region-dependent factors (e.g., prey availability) also play a role. In a broader context, our results show that simple physical effects of sea-ice changes on animal behavior (i.e., where and how efficiently animals walk, swim, rest, and hunt) can have substantial fitness consequences. Biological effects that occur at lower trophic levels (i.e., changes in prey field) are also important but are more complex and difficult to predict. Therefore, examining the physical effects of sea-ice changes on behavior for a range of polar predators (seabirds and marine mammals) using animal-tracking tools is a promising avenue for better predicting future changes in polar ecosystems.

## MATERIALS AND METHODS

### Fieldwork and instruments

Fieldwork was conducted at the Hukuro Cove colony (69.21°S, 39.63°E) in Lützow-Holm Bay, Antarctica, from late December to early February in four seasons (2010/11, 2011/12, 2012/13, and 2016/17). All the experimental procedures were approved by the Ministry of the Environment, Japan. One hundred seventy-five Adélie penguins, rearing one or two chicks, were captured using a dip net when leaving for a foraging trip, weighed and measured, and then instrumented (see below) before they were released. The instruments were recovered by recapturing the birds when they returned to their nests. Sex of the instrumented birds was inferred from an index based on bill length, bill depth, and flipper width (*35*); however, when two individuals making a pair were inferred as the same sex, the individual with the higher index was regarded as a male and the other individual as a female. By using tape, each bird was equipped with either (i) a GPS logger on the back, (ii) a GPS logger and an accelerometer on the back, (iii) a GPS logger on the back and an accelerometer on both the back and the head, or (iv) a video camera on the back and an accelerometer on both the back and the head (table S1). Three models of GPS loggers (GPL380-DT, 86 g, Little Leonardo; CatLog, 43 g, Catnip Technologies; CatLog2, 26 g, Catnip Technologies), two models of accelerometers (ORI400-D3GT, 9 g, Little Leonardo; AXY-Depth, 7 g, TechnoSmArt), and two models of video cameras (DVL100, 33 to 41 g, Little Leonardo; DVL400, 80 g, Little Leonardo) were used. The total weight of the instruments for each bird was 26 to 104 g, accounting for 0.6 to 2.4% of the average body mass of the instrumented birds (4.3 kg).

To determine the growth rates of chicks, 20 to 40 chicks were marked every season with a plastic flipper band in late December (when most chicks were <10 days old) and weighed every 5 days until late January or early February (when all surviving chicks formed an aggregation called a crèche). Growth rate (grams per day) was calculated as the slope of least-squares regression line for body mass plotted against calendar date. Some chicks died of starvation or predation by south polar skuas *Stercorarius maccormicki*, and those chicks were excluded from the growth rate analyses. Survival rates of the banded chicks (64% in 2010/11, 50% in 2011/12, 89% in 2012/13, and 95% in 2016/17) showed a similar trend to the breeding success of the whole colony (Fig. 2G). Breeding success, defined as the number of fledglings produced per nest, was calculated as the number of survived chicks in late January or early February (at the crèche stage close to fledging) divided by the number of active nests in late December. Stomach contents were collected from 8 to 11 birds each season using the standard stomach-flushing method (*36*), and prey species were identified. Mass-based prey composition was measured for each individual and then averaged across individuals for each season (Table 1).

In the ice-free season (2016/17), chlorophyll a concentrations of surface water were measured by a ship at four locations 17 to 21 km off the penguin colony. Water samples were taken from the water pumped through the hull (from approximately 9 m depth) and processed following (*37*).

### Tag data analyses

Data recorded by electronic tags were analyzed using the software IGOR Pro (WaveMetrics). Foraging trips were extracted from the GPS data as any return excursions from the nest with the maximum distance reached of >100 m and a duration of >1 hour. A subset of GPS tracking data that also had accelerometer records (i.e., three-axial accelerations, depth, and temperature) was used to estimate penguin energy expenditure during foraging trips. To do so, penguin activity was categorized into (i) diving (when depth was >1 m), (ii) walking (when GPS position was changing, body posture was upright, and the ambient temperature was air temperature), (iii) resting at the sea surface (when depth was zero, body posture was horizontal, and the ambient temperature was water temperature), and (iv) resting on land or ice (when GPS position was constant, depth was zero, acceleration fluctuated minimally, and the ambient temperature was air temperature) (fig. S3). Metabolic rates during diving, walking, resting at the sea surface, and resting on land or ice were set at 14.0 W kg^−1^ (*38*) [assuming a swim speed of 2.0 m s^−1^ (*39*)], 10.7 W kg^−1^ (*40*) (assuming a walking speed of 1.5 km hour^−1^ based on our GPS tracking data), 8.4 W kg^−1^, and 4.8 W kg^−1^ (*41*), respectively. These values came from experiments with Adélie or closely related gentoo penguins conducted during the summer in Antarctica; therefore, we assume that thermoregulation costs relevant to our datasets are included in the values. Activity-specific metabolic rates were multiplied by the time spent in that activity to yield activity-specific energy expenditures, and the sum of activity-specific energy expenditures over all four activities was our estimate of total energy expenditures during the trips (fig. S4).

Diving behavior and foraging success were examined for the individuals that carried two accelerometers (on the back and the head). Diving bouts composed of krill-feeding dives, which are readily detected by the characteristic depth profile with repeated up-and-down movements (*42*), were extracted, and dive depth and duration of each dive were calculated (Fig. 3). To characterize dive profiles, each dive was separated into descent, bottom, and ascent phases (fig. S5). The end of the descent phase (i.e., the beginning of the bottom phase) and the beginning of the ascent phase (i.e., the end of the bottom phase) were defined as the initial and last moments, respectively, when pitch angle of the birds turned from negative (downward) to positive (upward) at a depth greater than 50% of the maximum depth reached during that dive. Next, the number of krill captured in a dive, standardized for 100-s dive duration (close to the mean dive duration of krill-feeding dives, 105 s), was estimated. The timing of krill capture events was inferred on the basis of the difference between the vectorial sum of three-axial head accelerations and that of the body accelerations (*20*). The optimal threshold (0.25 g) was previously determined with the Little Leonardo accelerometers by linking the acceleration records to the simultaneously recorded video footages and was shown to produce a high detection rate (83%) and a low false discovery rate (15%) when the prey is krill (*20*). Only the Little Leonardo accelerometers were used in 2010/11, 2011/12, and 2012/13, but the TechnoSmArt accelerometers were used in 2016/17 for 7 birds in addition to the Little Leonardo accelerometers used for 12 birds. These two devices were experimentally shown to produce slightly different acceleration signatures for a given motion. For a fair comparison across seasons, data recorded by the TechnoSmArt accelerometers were excluded from this analysis. The number of krill capture events for each dive was divided by the dive duration (in seconds) and multiplied by 100 to account for the variability in dive duration. The dives without any krill capture events were excluded from the calculation of krill capture rates, because those dives may represent traveling. In this study, we focused on krill capture events and did not extract all prey capture signals from the acceleration data. This is because krill (*E. superba* and *E. crystallorophias*) is the most important prey in our study site based on the previous (*16*) and current (Table 1) stomach content analyses, although fish can also be important depending on seasons. Moreover, our penguin-borne video footage showed a variety of minor foraging events (e.g., catching bottom-living fish, amphipod, and jellyfish). These events are difficult to detect from acceleration records alone and would cause large errors if we extract all prey capture signals from non–krill-feeding dives.

### Statistics

The records of penguin behavior (trip duration, trip distance, krill capture rates, and dive duration) were compared among seasons by using linear mixed-effect models with season as a predictor variable and penguin ID as a random factor, using the software R with the extension lme4 (*43*). Trip duration, trip distance, and krill capture rates were log_10_ transformed due to their positively skewed distributions. When modeling dive duration, dive depth was included as a predictor variable. Statistical significance was tested by comparing the full models and the model without the season variable using the likelihood ratio test. Adult body mass and chick growth rates, where a single value was recorded for each individual, were compared among seasons using ANOVA.

## Supplementary Material

aba4828_Movie_S1.mp4

aba4828_SM.pdf

## SUPPLEMENTARY MATERIALS

Supplementary material for this article is available at http://advances.sciencemag.org/cgi/content/full/6/26/eaba4828/DC1
