# Supplementary material for: Foraging behavior links sea ice to breeding success in Antarctic penguins
Source: Sci Adv. 2020 Jun 24;6(26):eaba4828. doi: 10.1126/sciadv.aba4828 (PMC7314529; doi:10.1126/sciadv.aba4828)
Supplement: aba4828_SM.pdf [file aba4828_SM.pdf]

Science Advances
AAAS

advances.sciencemag.org/cgi/content/full/6/26/eaba4828/DC1

# Supplementary Materials for

## Foraging behavior links sea ice to breeding success in Antarctic penguins

Yuuki Y. Watanabe*, Kentaro Ito, Nobuo Kokubun, Akinori Takahashi

*Corresponding author. Email: watanabe.yuuki@nipr.ac.jp

Published 24 June 2020, *Sci. Adv.* **6**, eaba4828 (2020)
DOI: 10.1126/sciadv.aba4828

**The PDF file includes:**

Figs. S1 to S7
Table S1

**Other Supplementary Material for this manuscript includes the following:**

(available at advances.sciencemag.org/cgi/content/full/6/26/eaba4828/DC1)

Movie S1

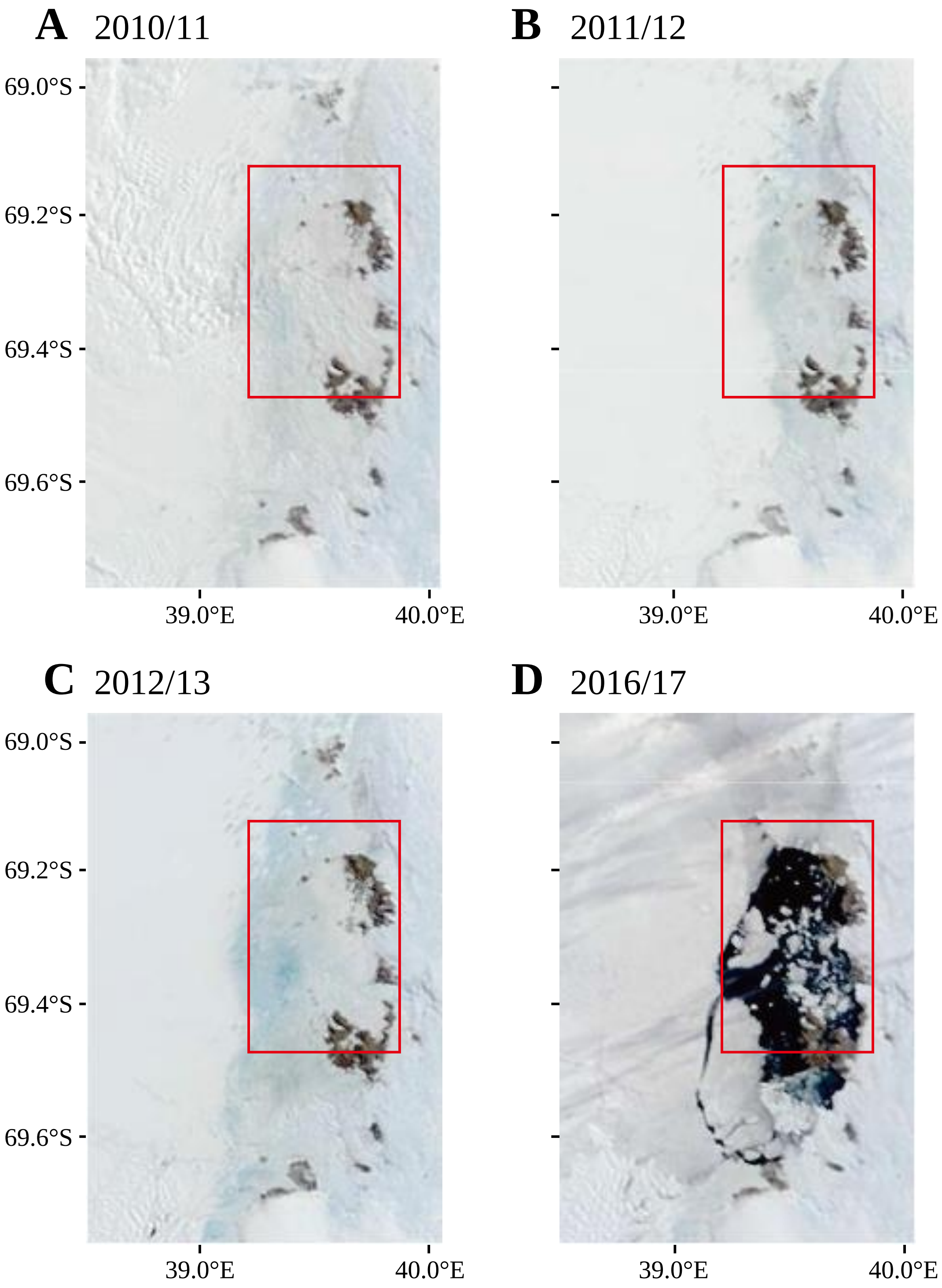


**Fig. S1. Satellite images of the study area in January of each season.** An extensive polynya is visible in (D). Red rectangles represent the area shown in Fig. 1, E to H. Images were obtained at the National Aeronautics and Space Administration (NASA) Worldview Snapshots (https://wvs.earthdata.nasa.gov).

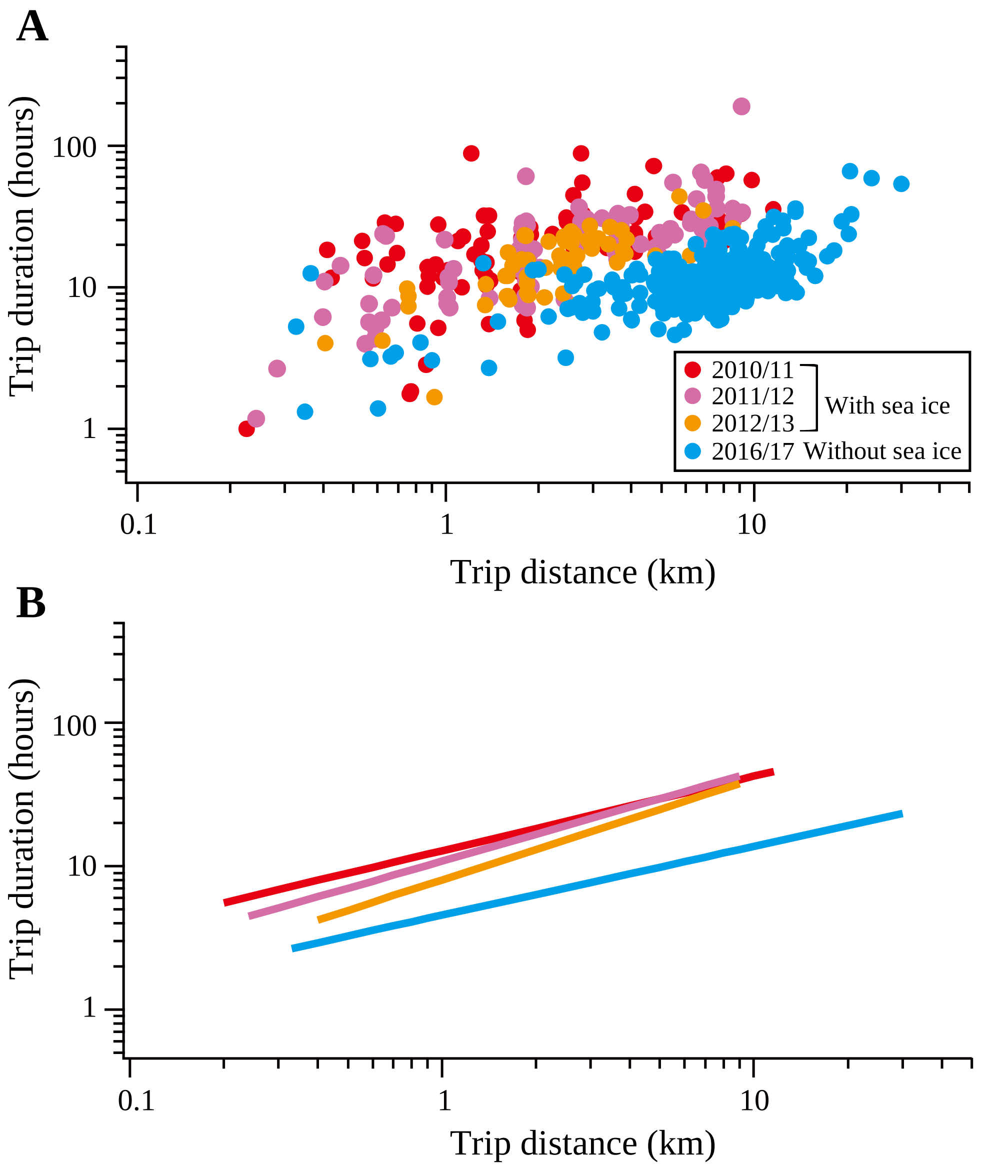


**Fig. S2. Relationship between trip distance and trip duration.** (A) Trips in different seasons are shown in different colors. (B) The ordinary least-squares regression lines (on log-log scale) are shown for each season (2010/11, $Y$=12.8×$X^{0.52}$, $R^2$=0.31, $N$=91; 2011/12, $Y$=10.1×$X^{0.68}$, $R^2$=0.59, $N$=92; 2012/13, $Y$=8.0×$X^{0.70}$, $R^2$=0.53, $N$=64; 2016/17, $Y$=4.5×$X^{0.49}$, $R^2$=0.33, $N$=260).

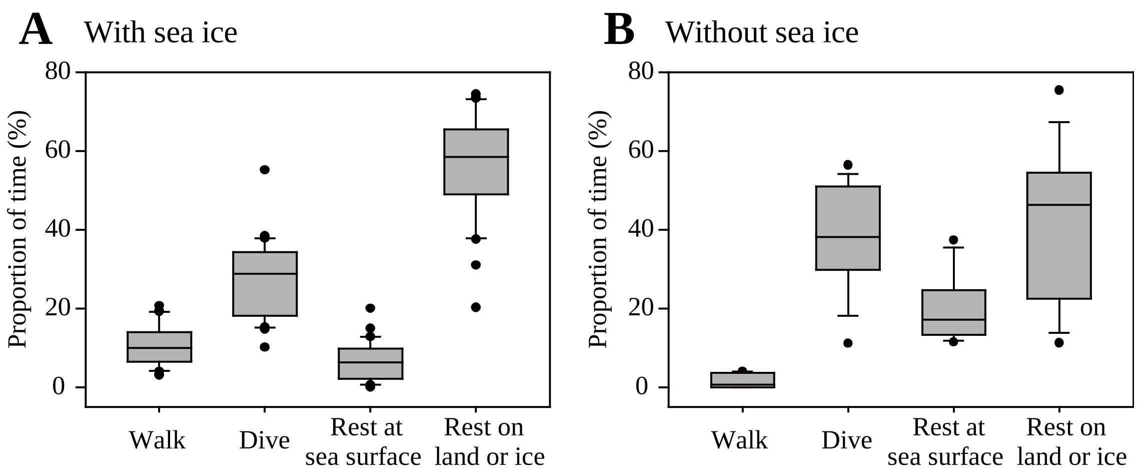


**Fig. S3. Proportion of time spent in each behavioral category during foraging trips.** Box plots for (A) ice-covered seasons, where the birds tracked in 2010/11 (*N*=12 trips), 2011/12 (*N*=9 trips), and 2012/13 (*N*=13 trips) are pooled, and (B) ice-free 2016/17 season (*N*=17 trips). The 25th percentile, the median, and the 75th percentile are shown by boxes, the 10th and 90th percentiles are shown by error bars, and the data points outside the 10th and 90th percentiles are shown by small circles.

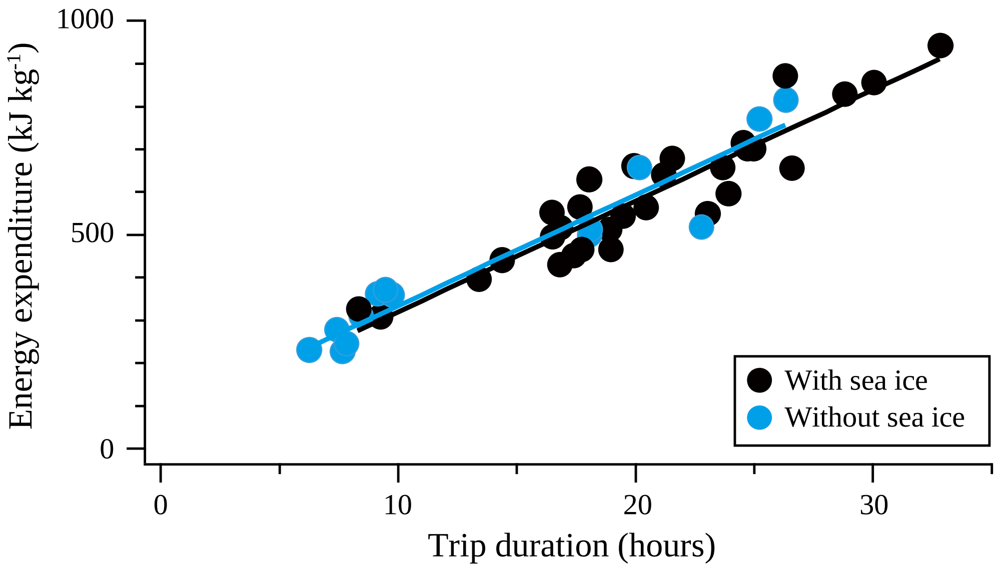


**Fig. S4. Relationship between trip duration and estimated energy expenditure during trips.** Ice-covered seasons (2010/11, 2011/12, and 2012/13) (black circles) do not differ significantly from ice-free season (2016/17) (light blue circles). Regression lines are based on linear mixed-effect models (ice-covered season, $Y$=26.0×$X$+60.0; ice-free season, $Y$=26.0×$X$+74.4).

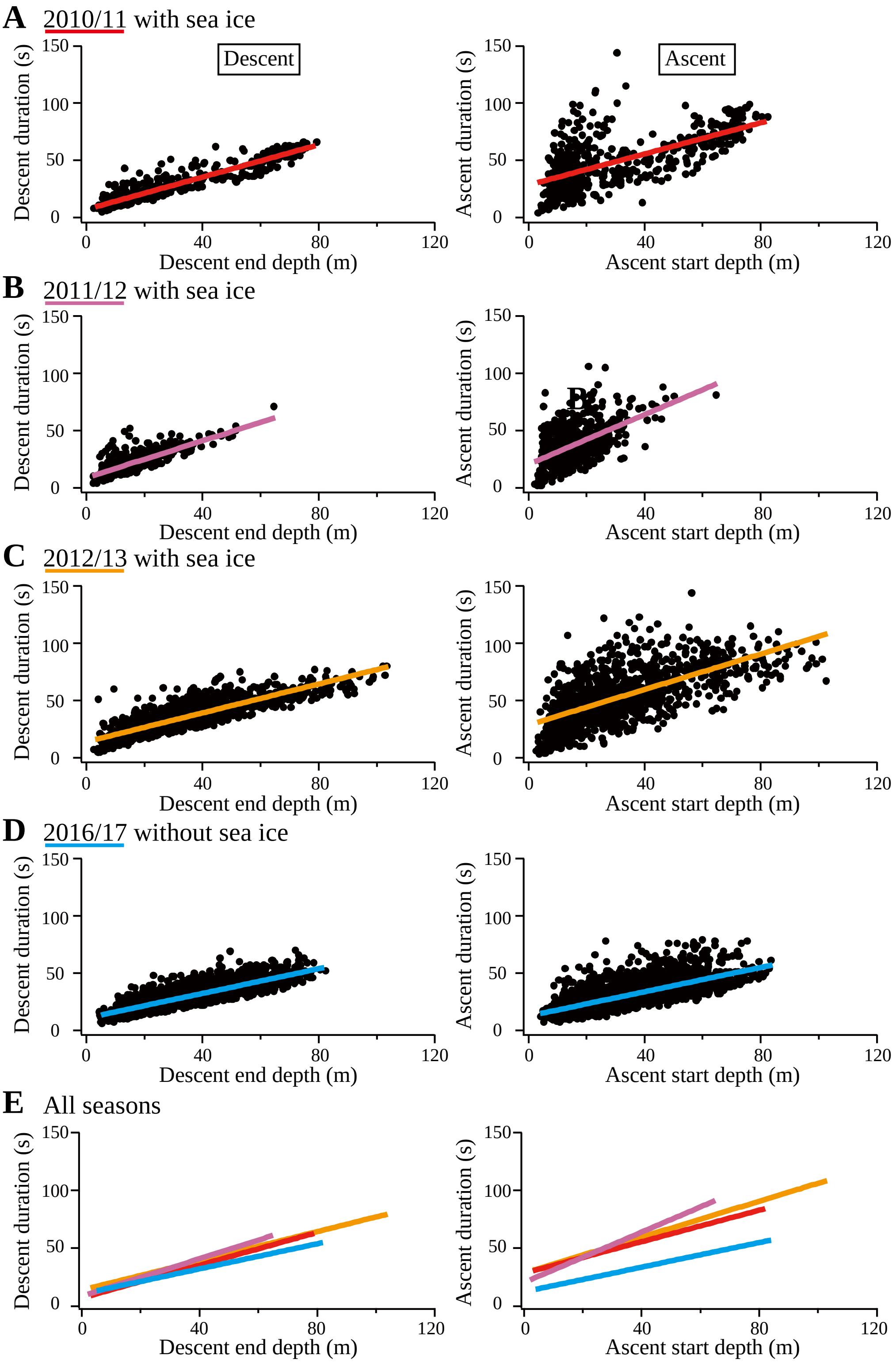


**Fig. S5. Relationship between vertical travel distance and duration of krill-feeding dives.** (A to C) Ice-covered seasons (2010/11, 2011/12, and 2012/13), (D) ice-free season (2016/17), and (E) interannual comparison are shown, with left and right panels

representing descent and ascent phases of dives, respectively (see Materials and Methods for definition). Descent phases have similar duration with similar variabilities for all seasons, whereas ascent phases have shorter and less variable duration for a given depth in the ice-free season compared with the ice-covered seasons. The ordinary least-squares regression lines are shown for descent phases (2010/11, $Y$=0.71×$X$+7.1, $R^2$=0.90, $N$=531; 2011/12, $Y$=0.80×$X$+8.8, $R^2$=0.60, $N$=485; 2012/13, $Y$=0.63×$X$+13.9, $R^2$=0.74, $N$=1119; 2016/17, $Y$=0.54×$X$+10.6, $R^2$=0.70, $N$=2275) and ascent phases (2010/11, $Y$=0.68×$X$+28.4, $R^2$=0.43, $N$=531; 2011/12, $Y$=1.09×$X$+20.4, $R^2$=0.29, $N$=485; 2012/13, $Y$=0.77×$X$+28.6, $R^2$=0.43, $N$=1119; 2016/17, $Y$=0.53×$X$+12.4, $R^2$=0.51, $N$=2275).

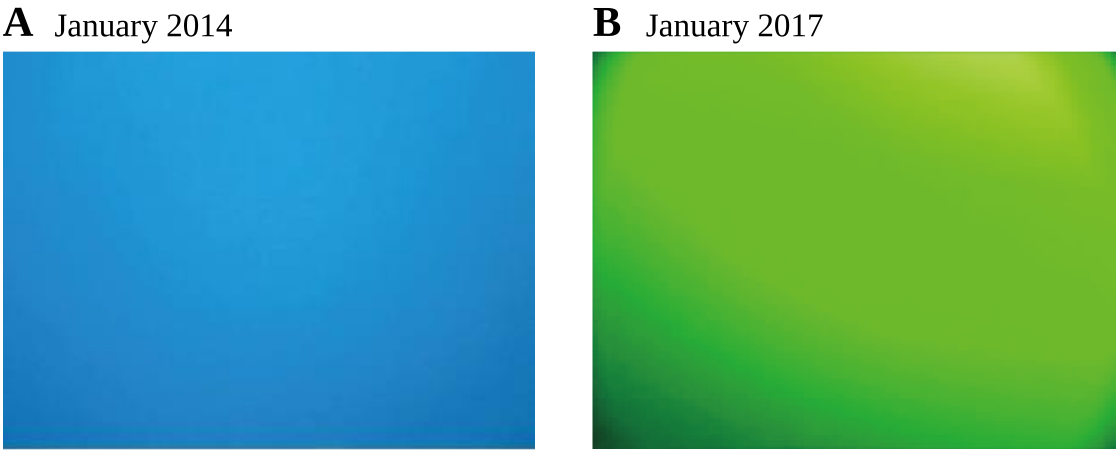


**Fig. S6. Different colors of the water.** Images captured from penguin-borne video footage filmed during the ascent phase of a dive in (A) January 2014, when sea ice was extensive, and (B) January 2017, when sea ice was nearly absent.

**A** *Euphausia superba* **B** *Euphausia crystallorophias*

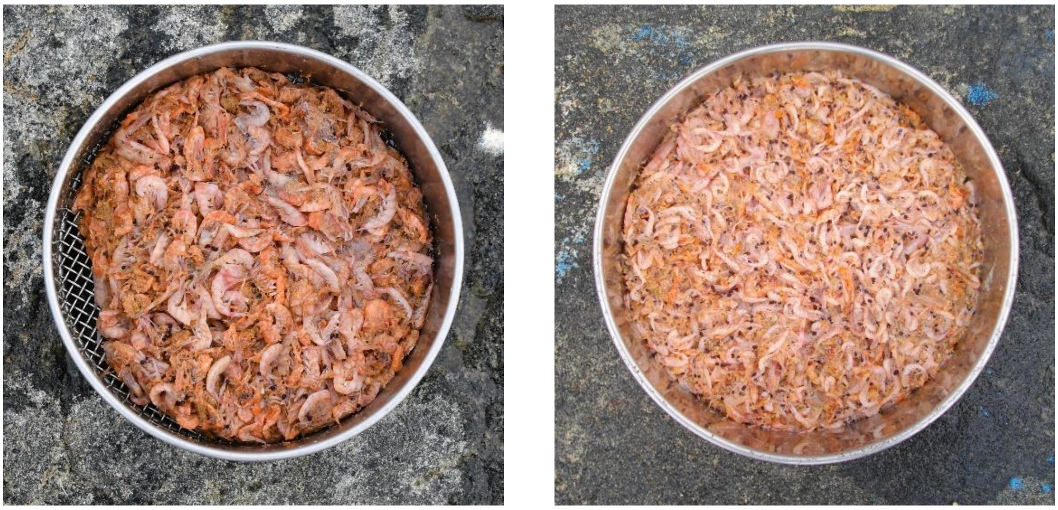

**Fig. S7. Krill sampled from penguin stomachs.** (A) *Euphausia superba* and (B) *Euphausia crystallorophias* sampled from a penguin in January 2017 and January 2011, respectively (Photo credit: Y. Y. W., National Institute of Polar Research). *E. superba* is larger than *E. crystallorophias*. The sieve is 24 cm in diameter.

**Table S1. Number of birds equipped for each season and each device category.**

| Season | GPS (back) | GPS (back) Accelerometer (back) | GPS (back) Accelerometer (back and head) | Video (back) Accelerometer (back and head) | Total |
|---|---|---|---|---|---|
| 2010/11 | 8 | 8 | 0 | 14 | 30 |
| 2011/12 | 12 | 0 | 16 | 8 | 36 |
| 2012/13 | 10 | 0 | 18 | 18 | 46 |
| 2016/17 | 17 | 10 | 11 | 25 | 63 |
| | | | | Grand total | 175 |
